# How Can We Hold Our Husbands? or Suggestions for the Correction of the “Divorce Evil”

**Published:** 1912-06

**Authors:** 


### TEXAS MEDICAL JOURNAL.

Established July, 1885

F. E. DANIEL, M. D., - - - - - Editor, Publisher and Proprietor

Published Monthly.—Subscription, $1.00 a Year.

VOL. XXVII. AUSTIN, JUNE, 1912. NO. 12.

The publisher is not responsible for the views of the contributors.

### Original Articles.

For Texas Medical Journal.

### How Can We Hold Our Husbands? or Suggestions for the Correction of the "Divorce Evil."

BY A WIFE AND MOTHER.

The cry of the hour is: "The divorce evil; it is spreading. How can we stop it?"

Being the wife of a physician, as well as the daughter of one, and having studied medicine to some extent, with the idea of becoming a physician myself, which plans were interrupted by marriage; and now, being the mother of four sons and three daughters, I am deeply interested in this subject and its cause and cure. The study of the human frame, together with its qualities of heart and mind, as well as ailments, is, as always, of keen interest to me, and never more vitally so than now, at the present day, with the responsibilities of maternity full upon me— and— "the signs of the times"—"The Divorce Evil"—What it is? Or; What *causes* it? and how can we stop it, or cure it?

Did you ever stand on the seashore, and, looking far out to sea, lose yourself in contemplation of the vastness of the deep, to be suddenly brought back to the present, or made aware of your surroundings, by your feet growing cold, and looking down, see that you were surrounded by the incoming tide? And, looking farther down the beach, see children, who will be caught in the undertow and swept out to sea, unless warned? Then you appreciate my position. I am standing on the beach, and the tide is very near me (for my children will soon be taking their places in the world), and I realize how strong the undertow (of temptation and sin) is, and they are not alone, and *I*, seeing, must warn them and others, also.

To my mind, there are but *two* ways to prevent the "divorce evil" from spreading: one is to "right about face" and be better wives ourselves; and the other is to warn our children and let them profit by, or rather give them the benefit of, our own experience.

You say at once: "Oh, that does not apply to *me*. I am as good a wife as I know how to be." That may be, madam, but are you *sure that you know how?*

Now, I propose to treat this subject without gloves—as woman to woman—and give the opportunity, right here, for any who do not wish to be shocked, or are too modest, to mention such things, to withdraw, at this point, as quickly as possible, so that we may proceed without interruption or unfair criticism.

I come to you, in sisterly affection, and would ask you to listen, in the same spirit, that we may be mutually helpful to each other.

How do we know, or what assurance have we, that next month or next year, we will not be in a divorce court ourselves? That *we can hold our own husbands?* Listen, "Let him who thinketh he standeth, take heed, lest he fall."

You may suddenly decide that you are "unequally yoked"—for the "tide is coming in," and *"the other man"* as well as the "other woman" is in the undertow.

The atmosphere we breathe is gradually changing, from so much pressure, for the spirit of the age is "free love," "free thought," "free speech" and "free action." The "free moral agency" doctrine is being accepted in the *letter* of the law, instead of in the *spirit,* and we are trying to live "moral" (man, or self-governed) lives, instead of "God-fearing," prayer-sustaining lives, as did our parents; and the "divorce evil" is on the increase, and yet we marvel!

Now, listen: "Marriage *is* a failure"—as prescribed and practiced by us and our social system. Now, this, the key-note of our nation—Marriage and the Home—being the cornerstone of our national greatness, *must be "reset."*

Just as we have outgrown and overcome superstition and dogmas in the past, so must we change *this,* the vital point of our superstructure. It must change to meet the times.

The "times" have changed, and we have changed—and the attitude of the nation, along all lines; but this vital point we refuse to alter, and permit it to become our national stumbling block, instead of the institution it was intended to be—our national cornerstone.

When the home and its attributes were destroyed in old Roman times, then dynasties fell, Corinthian columns and temples of fabulous wealth melted as if in a crucible, or crumbled and vanished as a dream.

Now, there are but two ways to correct the state of affairs: First, for those of us who are wives already to "right about face," and "charge the enemy" by having plenty of ammunition at home; and, second, rout her for all time by preparing our sons and daughters for the onslaught. I say *sons* advisedly, for I think the *men* of the future must be prepared as well as the women. The *men, today,* are as responsible for the "divorce evil" as the women. How? Why? Follow me, and I will try to show you.

Our own education was wrong, and we are educating *into* our boys and girls much that will have to be educated *out* of them later on.

Now, to you wives, first:

A wealthy, refined, cultured lady sought her family physician with this question: "Doctor, why is it that so many men, hitherto moral and upright, will, at the age of 50 or 60, suddenly become untrue to the wife they have loved and cherished for twenty-five or thirty years?" Her own husband having done this same thing, she was vitally interested. Her physician knew her to be regarded in all respects as a model wife. Her home and children reflected the care she had bestowed; yet, he (the doctor) asked bluntly: "Have you done your *best* to hold your husband?" "Have you been an ideal wife?"* The deeper meaning underlying his question is the *key* to the whole situation.

A man has but *one* nature. He is passionate and aggressive. This must be right, for God made him "in His own image," and pronounced His work "good."

Woman has *two* natures, passive and receptive. She was made *after* man, to be his complement. She was made for man's comfort, and, therefore, his other self. This must also be right, for, after making man, God saw that it was not good for him to be alone; *by himself he was incomplete.* He could not reproduce his kind; for he represented but half a plan. It was necessary to provide a counterpart, or complement, that the two halves might make a complete whole, thereby, the joining together of one nature to the other, a perfect union was made. Just as we say today, "it

*Dr. Bogart in Texas Medical Journal.

takes two halves to make a whole," so marriage, or its fundamental principle, the union of the sexes, was sanctioned by God, while the *form* of that function, or outward manifestation of this agreement between the sexes, was left to the dictates of society.

Now, you say: "What has this to do with the divorce evil?" Everything. I think our attitude or understanding of our responsibility as wives to the marriage relation is the *whole cause,* or the key to the whole situation. It is not so much a question of "how shall we stop the divorce evil?" I think, as *how shall we hold our husbands?* As I said awhile ago, the tide is *coming in,* and the "other man" and the "other woman" are in the undertow.

In the first place, *all men are passionate.* That is their divine right, but *not every* woman may stimulate or arouse their passion.

A wife said to me once: "I just *loathe* my husband; he is such a *beast.* Why, do you know, I can't go near him, nor caress him, that he does not make demands of me, and *I* am just the other way; I *don't care for it at all.*" Poor fool. She gloried in "not caring for it at all," and pitied herself as a martyr (the world is full of her kind). That's what's the matter with the *divorce* question. But, *I* say, *poor man;* deprived of his marital rights, conjugal happiness, and tied to *a clam for life!*"

And is he to blame, then, if he goes where the "red light" burns, because he is attracted by its warm glow?

That's what's the matter. Too many wives are *passive,* not *active* or responsive.

What does the Good Book say about "luke warm Christians"? It says, it "would spew them out of its mouth." Yet luke warm *wives—women who are wives only in name,* but not in the spirit, we tolerate and uphold as martyrs, and go on trying to *legislate against* prostitution. What do the thinking minds say: "You *can never cure* or stamp out prostitution *by law* or enactment of laws, for *as long as there* is the *demand,* there will be the *supply.*"

There would be no *"red light"* districts; there would be *no divorces;* there would be *no polygamy* in our land, nor any need of it *if wives did their full duty by their husbands!*

Nor would there be any need of suffrage, nor of any one to espouse its cause, save in the ranks of old maids and widows, perhaps, if *wives* were *wives* in the Bible sense; for women would be too busy at home, on the one hand, and have no need of "rights," on the other hand, for a man's "mistress," proverbially, "gets all she *wants,*" from him; then why not his wife? *I tell you she would, if she were alive to her duties.*

I say our social system is wrong, and our education was wrong, or we would never have been confronted with this evil.

*"A man goes after the woman who offers the most to his nature,"* was said by a wise woman.

Now, instead of a wife feeling *abused* and loathing her husband because he admires her *for herself,* she should feel *complimented* that *she,* above all women, can attract him, for she is a god, and he worships at her shrine. Ah, woman, did you ever look up, through your conjugal relations, instead of down? Try it. I think it is every woman's privilege, even *duty,* to do this. But more of the *duty* later.

Now, do you know that as men grow older they become more amorous? This probably accounts for the maxim, "No fool like an old fool"; and, also, why so many *old* men marry very young girls. This may be accounted for in two ways: First, because of man's innate vanity, and, second, by his natural physical condition. As a man grows older his mental powers become more sluggish, and his animal nature predominates, and his desire for the opposite sex is thus stimulated by two causes: First, his desire to show that he is "still a man," by being attractive to the opposite sex—sufficiently so to be accepted as a sutor. This flatters his vanity. And, then, in conformity to nature, which provides that the loss or failure of one sense is compensated for by the quickening of others. Inasmuch as the mind is usually the first weakened by age, so the other functions of the body are stimulated in proportion as that function wanes. This accounts for medical records of idiots and the insane being addicted to masturbation; and invalid men, especially tubercular, being the most passionate of all men.

On the other hand, women go through a *physiological change* at about 40, and we note that they are most active, mentally, from 40 to 60 years. This is borne out by the biographies of women writers, etc. Now, when this physical change comes upon women their sexual desire diminishes in proportion to their mental activity; and many women who are not inclined to literature devote their energies at this time to church, club or social duties; studiously avoiding the marriage relation, saying to their husbands: "We are too old, now, for such things." And he, abashed at the rebuke, or in pity for her state of health, goes to some one more sympathetic, and thus we have the husband of 50 becoming the town roué.

Oh, woman, in the glory of your womanhood! Oh, wives, in the sublimity of your wifehood! Listen to me: Better be a *self-*

*sacrificing* wife than a *sacrificed one,* for you will be the one or the other; it is inevitable.

Now, as to why so many—or I will say the majority of—wives are passive, or as physicians say, "are lacking in sexual feeling." This arises from two causes, I think. *False education* or the misconception of what a true marriage really is, and *early marriage.*

These are the two principal causes, and they produce two subcauses or deterrent causes: Fear of pregnancy and mismating.

In the first place, our girlhood education was wrong. How far, I can best show by contrast.

We were taught to keep that instinct down. "It is unholy," "impure." A boy was taught that "it is natural," "glorious." The girl was told "it is for *procreative* reasons," alone, and must not be thought of or mentioned. A boy was taught that through it he was to derive his greatest pleasure and gratification, and as he grew older, the more he cultivated it, the happier he would be. In fact, as one man expressed it: "It is all there is in life." "The gratification of this" dominant "trait" or characteristic "is as natural as eating and sleeping and vastly more satisfying." Hence, to a man, it constitutes his "pursuit of happiness," and is to him first and last everything—all else in life being secondary to it.

A girl is taught modesty of habit, as well as speech and conduct. To neither *think* nor *speak* of these things; they are impure, and her mind *must be pure.*

Now, by contrast, I think, you can see wherein lies the difficulty which produces a never-ending conflict. Take a gentle, pure, refined girl, and a strong, healthy man, with their training thus at variance, and place them in a room, in the relation of bride and groom and wait the result.

Her training of a lifetime must be mastered in a moment; her *nature* must change to meet the situation, bravely, smilingly; and *if she loves the man* (for his sake) she will soon conquer the first shock to her maiden modesty, and the prejudices of her nature and of a lifetime, and will soon pass from the *passive,* or submissive, to the *active* or receptive.

Now, many women, I believe, *never* pass out of the first *state,* because they are married in the sight of the law, but *not mated.* *To be married and not mated* is believed to be a cause for divorce now, and divorce is on the increase simply because women are beginning to see that their early education was wrong, and that the sexual life is a part of their lives, as much as that of the man, and they are getting bold enough to think it right to break a yoke instead of bending under it. Now, this is why early marriages *are not* advisable. A girl is not fully matured *in mind,* if in body, before 22 or 23. The first eighteen years of her life is spent in physical development, and she *has not* mature judgment, or a *live,* active mentality under that age. Hence, if a girl waited to be 22 or 23 she would come nearer choosing wisely, as a man does, for *physical* reasons.

This immaturity of judgment is the primal cause of early marriage, with attendant influences of outside causes, in which the girl is the victim, and is the prime cause of so many separations and divorces in later life, when you would expect more sense. But they have put up with each other until the "other man" (or woman) is found, the one who really answers the call of their half-starved nature, and they (emboldened by the spirit of the times in which we live) immediately revolt from, or rebel at, the chronic state of "putting up with" each other, which has been going on for so many years.

Now, women, listen! This brings me to my two sub-causes of the passive state, or indifference of wives. The first cause, I think, is mismating. This realization that they can not respond to their husbands is due most often to the fact that they are mismated; that is, that *he* is *not* the man who calls to her nature. Enjoyment of sexual life is as natural in one sex as in the other. This is demonstrated by appliances for women for their gratification found in China, and other foreign countries, to be used by them when confined in harems or other places where they are deprived of the natural caresses of man.

Medical science has not determined whether the seat of passion is in the spinal cord or in the brain. However that may be, *mind* plays an important part in this act, as in everything else we do.

I think there is good in everything, if we but look for it. Now hear what a Christian Scientist told me. This little woman, a pure, true wife and mother, in speaking to me of her infelicity, said: "Do you know that for a long time I could not bear his caresses, until I finally determined to see him, in my mind, as beautiful as Apollo, and you don't know what a change it has made in me."

Now, this good, pure, true little wife, struggling along with her duty, blinded by the superstition of ages, had suddenly come into the light, yet failed to see that within her hand was the key for which other wives have been struggling.

She, in her suffering, had arrived at the door, through which, now, all other wives may pass. And, wives, let me tell you, it is your remedy, if you are one of the ill-mated ones, not the divorce court and publicity, but "close thy door and pray to thy Father in secret and He will reward thee openly." When your husband approaches you, instead of shrinking from him, see in him the image you would have him be, close your eyes if necessary, but fix your mind, and before long you will be happy to see the transformation in yourself and the renewed joy it will bring to him. It will more than recompense you for your secret endeavor.

The second sub or deterrent cause of why a wife does not, or will not, yield herself entirely to her husband's pleasure is the fear of conception.

Now, in the first place, procreation is the Divine consummation of marriage.

Woman was made, not man, to propagate the species. That is why love of children is stronger in her than in him. And when you deny yourself child-bearing, which is as natural a function of your body as menstruation, or anything else, you starve that part of your nature, which in turn starves, or presses or pinches some other part of your being. This, then, manifests itself *outwardly* by irritability prompted by an unsatisfied longing, which you can not describe. The prevention of pregnancy is one of the most fruitful causes of disease of the pelvic organs. Why? Because nature has been tampered with, if not outraged, and she rebels. Sit for an hour, in any physician's waiting room, and see who form his clientele. The largest per cent are childless women, or the mothers of only one or two children, and old maids. You rarely find the mother of a large family there.

We hear much now-a-days about "limiting the family," etc. "It should be *quality* and not *quantity*," etc. The "high cost of living" being a barrier, etc.

I am heartily in favor of sterilizing the *unfit*, permitting only the "fit" to marry, by demanding health certificates, etc., but I *do* say that with these regulations the "fit" will have to bear the brunt of populating the world, or in a few years we will find ourselves where France was when the cry went up, "France needs mothers!"

It is no harder to rear a large family today than it was in our grandmother's time; it is simply the lack of ability to meet conditions. If women spent more time in the home, studying "domestic science" and "economy," and less in "bridge" and discussing the equality of the sexes, the big families would be just as easily reared.

In this paper I will not attempt to show the benefits derived by a mother from child-bearing (in itself) to her, physically, mentally and morally, nor the advantage gained through it to both parents, and thus through the home circle to the State; but will close with a few thoughts as how best to help our children to profit by our mistakes and thus prevent divorce and its cause in the future.

First, let us modify the teaching of the sexes. Teach our daughters when they arrive at the age of puberty what the marriage relation really is, by citing our own experience, which will cement a bond of sympathy between mother and child. Next, that it is a *holy instinct*, and the gratification of it should be a sacred duty, not to be regarded lightly nor forgotten, but to assist them in mating for life.

We do not force our daughters into loveless marriages for money or social position any more, but we still fail in parental duty by not teaching them that this function is the key-note to their future happiness, and is the quintessence of Love. We should advise them *not* to marry under any consideration until they meet the man whose nature calls to theirs.

Tell them not to be impatient nor mistaken by social position, nor wealth, nor pleasing personality; those are charms only, and will vanish, unless suggested by that deeper feeling, that *true*, dominant love which recognizes no flaw in its affinity, but whose soul meets soul.

Teach our sons that they are gods, not beasts. Teach them more modesty and consideration of another's feelings.

Teach them that they are endowed with "passions as glorious as the sun" for divine reasons; and it must be the study of their lives to respect those reasons.

Could we be wives and mothers like this, then, indeed, would our children say of us, "She hath done what she could"; and would rise up and call us blessed; and our husband's encomium would be: "Age can not wither her, nor custom stale her infinite variety."

